# Decoding coronary artery calcification: metabolic reprogramming features and a promising circulating biomarker PXDN

**DOI:** 10.3389/fcell.2026.1714146

**Published:** 2026-04-10

**Authors:** Yi Lu, Hongli Wang, Miao Li, Zheng Wan, Junting Dai, Yanchun Ding

**Affiliations:** 1 Department of Cardiovascular, The Second Hospital of Dalian Medical University, Dalian, Liaoning, China; 2 Department of Pharmacy, The Second Hospital of Dalian Medical University, Dalian, Liaoning, China

**Keywords:** cardiovascular disease, circulating biomarkers, coronary artery calcification (CAC), metabolic reprogramming, PXDN

## Abstract

**Background:**

Coronary artery calcification (CAC) is a strong predictor of cardiovascular disease, yet metabolism-related molecular alterations underlying CAC remain poorly understood. This study aimed to explore metabolism-related transcriptional changes associated with CAC and identify potential circulating biomarkers for early detection and risk stratification.

**Methods:**

Two transcriptomic datasets, GSE58150 (discovery cohort) and GSE211752 (validation cohort), were analyzed after excluding poor-quality samples. Differential expression, gene set enrichment analysis (GSEA), and metabolic feature identification were performed. Weighted gene co-expression network analysis (WGCNA) identified CAC-related gene modules, whose functions were explored by GSEA. Differentially expressed genes were further evaluated via protein-protein interaction networks and LASSO regression to screen biomarker candidates. Diagnostic value was assessed by ROC curves, and regulatory networks of transcription factors and miRNAs were constructed. A CAC mouse model was then established, with qRT-PCR, Western blotting, and immunohistochemistry used to validate biomarker expression.

**Results:**

The discovery cohort included 8 CAC and eight controls; the validation cohort comprised 6 CAC and six controls. A total of 138 genes were upregulated and 104 downregulated in CAC. Functional enrichment indicated alterations in amino acid and vitamin metabolism. WGCNA highlighted the ME2 module as strongly correlated with CAC. GSEA revealed activation of immune-related pathways and suppression of metabolic pathways. LASSO regression identified five biomarker candidates: NRG1, PXDN, ACTL7A, ACSS3, and SHANK3. Among them, PXDN showed the strongest diagnostic performance (AUC 0.95 and 0.83 in discovery and validation cohorts, respectively) and higher expression associated with increased CAC risk. Regulatory analysis suggested PXDN may be modulated by multiple transcription factors and miRNAs. *In vivo*, PXDN mRNA and protein were significantly elevated in blood and coronary artery tissue of CAC mice.

**Conclusion:**

CAC is associated with transcriptional alterations in metabolism-related pathways. PXDN was identified as a potential circulating biomarker for CAC, providing a basis for future mechanistic studies and non-invasive risk assessment.

## 1 Introduction

Coronary artery disease (CAD) is a major cause of mortality on a global scale. According to the latest statistics, approximately 254.28 million people were diagnosed with CAD in 2021, with 8.99 million deaths attributed to the disease (Martin et al., 2025; Qu et al., 2023). Moreover, it is estimated that the prevalence of CAD will increase by 90% and mortality rates will rise by approximately 73.4% between 2025 and 2050 (Chong et al., 2024). The onset of CAD is associated with lipid dysregulation and local inflammatory responses (Gk, 2005; Weber and Noels, 2011). In the late stages of the disease, it often manifests as the formation of atherosclerotic plaques. Coronary artery calcification (CAC) has been demonstrated to be a valuable predictor of cardiovascular risk, as indicated by the findings of numerous preceding studies (Greenland et al., 2010; Detrano et al., 2008; Polonsky et al., 2010). These studies have shown that CAC is closely associated with an increased risk of myocardial infarction, stroke, and cardiovascular mortality. CAC has been shown to serve as an independent predictor of CAD risk (Greenland et al., 2010; Detrano et al., 2008; Polonsky et al., 2010). Among these, CAC detection and quantification using computed tomography (CT) have demonstrated high positive predictive values for CAD events and are commonly used tools for clinical assessment (Greenland et al., 2010). However, due to radiation exposure, high examination costs, and limited screening efficacy in asymptomatic patients, the clinical benefits of CAC scoring are very limited (Martin et al., 2025; Leong et al., 2017). Consequently, there is an imperative to identify novel, accessible, and highly sensitive biomarkers that will facilitate the early detection, dynamic monitoring, and precise risk assessment of CAC, thereby enhancing the management of CAD.

Metabolic reprogramming is defined as the adaptive alteration of energy and material metabolic pathways in cells under specific pathological or physiological conditions (Fernandez-Caggiano and Eaton, 2021; Algoet et al., 2023; Peng et al., 2021; Wang et al., 2024). A growing body of research has indicated a close association between metabolic reprogramming and the onset and progression of CAD (Chen X. et al., 2025; Li M. et al., 2025; Chen L. et al., 2025). Among these, abnormal calcium-phosphorus metabolism in vascular endothelium leading to phosphate deposition is an important pathological mechanism underlying CAC. The calcimimetic agent cinacalcet effectively inhibits the synthesis and secretion of parathyroid hormone (PTH), thereby reducing serum phosphate levels and alleviating CAC and cardiac fibrosis (Jono et al., 2000; Jaume et al., 2010; Tsuruta et al., 2008; Floege et al., 2010; Wu et al., 2014). Additionally, metabolic reprogramming of immune cells, particularly metabolic changes in macrophages, effectively promotes their transformation into pro-inflammatory phenotypes, exacerbating local inflammation and ultimately leading to tissue damage and vascular calcification (Makows et al., 2020; Gordon and Plüddemann, 2017; Chen et al., 2019; Chen et al., 2024). Furthermore, metabolic reprogramming has been demonstrated to affect the synthesis and degradation of substances such as fatty acids and amino acids (Duan et al., 2022; Zhang et al., 2019). This, in turn, has been shown to disrupt the biological pathways in which these substances participate, ultimately inducing vascular thickening and remodeling. However, current research on metabolic reprogramming in CAC is largely limited to single aspects, lacking comprehensive exploration of different metabolic pathways, especially the complex metabolic network alterations involved, which remain to be further elucidated.

Circulating biomarkers are defined as molecules present in blood or other bodily fluids that reflect specific physiological or pathological processes. These molecules may include proteins, metabolites, or nucleic acids. The significance of these biomarkers in various diseases is well-documented. For instance, troponin and B-type natriuretic peptide (BNP) are extensively utilized for the diagnosis and prognosis assessment of acute coronary syndrome and heart failure, respectively (Wong and Tse, 2021; Mansour et al., 2025; Nazir et al., 2025; Xiao et al., 2025). In a similar manner, circulating biomarkers have been successfully employed in oncology for auxiliary diagnosis and monitoring treatment responses. However, the application of circulating biomarkers in CAC remains limited. Given the central role of metabolic abnormalities in the development of CAC, circulating biomarkers based on metabolic reprogramming hold promise as non-invasive, cost-effective, and dynamic tools for disease assessment. Therefore, this study aims to comprehensively analyze the metabolic reprogramming characteristics associated with CAC and identify circulating biomarkers with potential diagnostic value, thereby providing a basis for early detection and personalized risk stratification of CAD.

## 2 Materials and methods

### 2.1 Data collection and pre-processing

The transcriptomic data utilized in this study were obtained from peripheral blood of participants and uniformly downloaded from the NCBI Gene Expression Omnibus (GEO) database (https://www.ncbi.nlm.nih.gov/geo/). GSE58150 (Sen et al., 2014) functions as the discovery cohort, comprising RNA-seq data from eight CAC cases and eight controls (matched for sex, age, and ethnicity). The validation cohort, GSE211752 (Heuschkel et al., 2022), consists of microarray data from six CAC cases and six controls.

Quality control (QC) was conducted separately for each dataset. For RNA-seq samples, those with total read counts <10 million or mitochondrial gene proportions >20% were excluded, and principal component analysis (PCA) was used to identify outliers, defined as samples located beyond 3 standard deviations from the group centroid. For microarray data, QC included inspection of intensity distribution (boxplots), relative log expression deviation >0.2, and PCA-based outlier detection (>3 standard deviations from the centroid).

For GSE58150, raw read counts were normalized based on gene length and sequencing depth, then converted to million transcript counts (TPM) values to enhance inter-group comparability. Correspondingly, raw CEL files for GSE211752 were downloaded and processed using the Robust Multi-array Average (RMA) algorithm, including background correction, quantile normalization, and log2 transformation. Gene annotation was performed using clusterProfiler (v.4.8.2) (Love et al., 2014) and AnnoProbe (https://github.com/jmzeng1314/AnnoProbe/) for GSE58150 and GSE211752, respectively. Probes mapping to multiple genes were excluded, and average expression values were calculated for genes with multiple probes. Discovery and validation analyses were conducted independently within each dataset rather than by merging cross-platform expression matrices, thus not accounting for batch effects.

**FIGURE 1:**
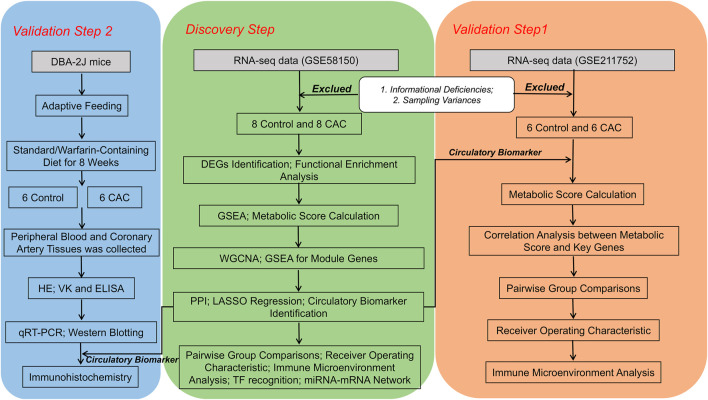
Flowchart of the workflow. Abbreviations are defined as follows: coronary artery calcification (CAC), differentially expressed genes (DEGs), enzyme-linked immunosorbent assay (ELISA), gene set enrichment analysis (GSEA), hematoxylin and eosin (HE), least absolute shrinkage and selection operator (Lasso), protein-protein interaction (PPI), quantitative real-time polymerase chain reaction (qRT-PCR), transcription factor (TF), weighted gene co-expression network analysis (WGCNA).

This study utilizes data from publicly available databases. Figure 1 depicts the flowchart of this study.

### 2.2 Functional enrichment analysis

Perform functional enrichment analysis on GSE58150 cohort. RNA-seq data (Counts value) was analyzed using the DEseq2 (v.1.40.2) (Wu et al., 2021). Free nucleic acids in peripheral blood (such as circulating cell-free DNA and RNA) primarily originate from cells undergoing apoptosis or necrosis, exhibiting low concentrations and complex background noise. To ensure sufficient genes for subsequent analysis, genes meeting the criteria of *P < 0.05* and ∣log2FC∣ ≥ 1were defined as differentially expressed genes (DEGs). Simultaneously, the Benjamini–Hochberg method was applied to all DEGs for genome-wide false discovery rate (FDR) correction. Detailed results were presented in Supplementary Table S1.

In order to identify potential biological pathways that may induce CAC, clusterProfiler (v.4.8.2) (Love et al., 2014) was utilized to perform over-representation analysis (ORA) and gene set enrichment analysis (GSEA) (Ritchie et al., 2015) on DEGs. The focus was on Gene Ontology (GO) and Kyoto Encyclopedia of Genes and Genomes (KEGG), where *P < 0.05* indicates that the signaling pathway is significant.

### 2.3 Metabolic score calculation

Two independent datasets (GSE58150 and GSE211752) were analyzed using two different sets of metabolic feature genes and the “ssGSEA” algorithm for metabolic score calculation. The following steps were taken in this process: (1) The datasets were normalized; (2) The metabolic feature gene sets built into IOBR (v.0.99.8) (Subramanian et al., 2005) were used to calculate the relevant data; (3) Metabolic-related genes were selected from the WikiPathway (https://www.wikipathways.org/) and REACTOME (https://curator.reactome.org/) databases for calculation; (4) Metabolic pathways with consistent trends in both datasets must be extracted for visualization.

### 2.4 Weighted gene co-expression network analysis

The weighted gene co-expression network analysis (WGCNA) was constructed using the “WGCNA” (v.1.69) (Zeng et al., 2021) to identify the central module associated with CAC. The process was carried out in the following steps: (1) Top 3,000 median absolute deviation (MAD) genes between samples from the GSE58150 were selected as the input dataset; (2) Outliers were removed using the “goodSamplesGenes” function; (3) Set the R^2^ cutoff value to 0.85 and use the “pickSoftThreshold” function to determine an appropriate threshold ensuring a scale-free network topology; (4) Plot the scale-free network’s exponent plot and average connectivity plot to validate threshold sensitivity; (5) The correlation matrix was transformed into a topological overlap matrix (TOM), which provides a more accurate reflection of gene co-expression; (6) Modules were identified through dynamic tree-cutting, and their relationship with CAC was assessed using module-trait relationships; (7) The module with the highest pearson correlation coefficient was selected as the central module; (8) Employ the “modulePreservation” function to conduct Zsummary-based WGCNA module stability analysis; (9) Central module genes were extracted and performed GSEA.

### 2.5 Protein-protein interaction and correlation analysis

To explore potential interactions between genes, the stringAPP plugin of Cytoscape (v.3.10.1) (Li et al., 2022) was utilized to construct a protein-protein interaction (PPI) network with the central module genes as input. In accordance with the network topology, only genes with degree value of more than five were retained. The visualization mode of the network was then adjusted according to the degree value, with a view to highlighting the importance of the genes. Furthermore, the genes were extracted from the network and pearson correlation coefficient was used to calculate the correlation between gene expressions. The results were presented as a heatmap, with *P < 0.05* indicating a reliable correlation.

### 2.6 Key gene identification

Least absolute shrinkage and selection operator (LASSO) regression was carried out using the glmnet (v4.1-3) on the gene expression matrix to identify candidate biomarkers. Patient classification served as the dependent variable in the regression framework. A penalization technique was implemented to mitigate overfitting and determine the most informative variables, with optimal lambda values selected according to the one-standard-error rule (lambda.1se). The subset of genes retained after regularization was incorporated into the prediction model, and a nomogram was used to visualize the contribution of the gene subset to the model.

The expression differences of gene subsets between groups were analyzed in two independent cohorts (GSE58150 and GSE211752). To validate the predictive efficacy, receiver operating characteristic (ROC) analysis was performed. The area under the ROC curve (AUC) was calculated using the pROC (v1.0-11) to assess the discriminatory power. Given the limited sample size, a bootstrap resampling procedure (R = 1000) was further conducted for candidate genes using the “boot” package (v1.3-31), and the 95% confidence intervals (CIs) of AUCs were estimated based on the resampled distributions. In addition, permutation tests were performed for each candidate gene to evaluate whether the observed AUCs were significantly greater than expected by chance. Finally, key genes were determined by integrating the AUC values, their bootstrap-derived 95% CIs, and the permutation test results.

### 2.7 Immune microenvironment analysis

In order to achieve a more sophisticated multidimensional analysis, the study employed three distinct computational models to decode the immune microenvironment (IME) across two independent cohorts using deconvolution algorithms. Specifically, xCell (Shannon et al., 2003) utilizes a single-gene set enrichment analysis algorithm, TIMER (Aran et al., 2017) employs a least-squares fitting model, and MCP-counter (Li et al., 2020) depends on a linear regression model. It is important to note that these three algorithms have been extensively applied to non-tumor peripheral blood sequencing data, and robust predictive performance has been demonstrated (Aran et al., 2017; Xu et al., 2025; Li B. et al., 2025; Hoffmann et al., 2024).

The deconvolution results were then visualized as cell type-specific immune infiltration scores, which had been uniformly normalized to enhance comparability. Subsequently, the “limma” algorithm was employed to analyze differences in cell type-specific immune infiltration scores between groups, with standardized results visualized as heatmaps. *P < 0.05* indicates a significant difference in the infiltration abundance of that particular cell type between the groups.

### 2.8 Transcription factor recognition and miRNA-mRNA network construction

In order to identify potential regulatory factors, transcription factors and miRNA-mRNA network were identified for key genes, specifically including: (1) The integration of five independent transcription factor databases (hTFtarget, ENCODE, TRRUST, GTRD, ChIP_Atlas, JASPAR (*FIMO_JASPAR and PWMEnrich_JASPAR)*) was undertaken, with the objective being the identification of transcription factors corresponding to key genes as targets. Furthermore, the extraction of transcription factors that exist simultaneously in the database was conducted for the purpose of constructing their regulatory network for key genes; (2) The key genes were used as input for the miRWalk (http://mirwalk.umm.uni-heidelberg.de/) website, with the objective being the identification of corresponding miRNAs and the construction of miRNA-mRNA network using Cytoscape (V3.10.1).

### 2.9 Animal model of CAC

Male DBA-2J mice, 7-8-week-old and weighing 18–20 g, were purchased from SPF (Beijing Biotechnology Co., Ltd, Beijing, China). To establish a model of CAC, mice were randomly assigned to either a control group or a CAC group, with six mice in each group. Mice in the CAC group were administered a warfarin-containing diet (3 mg/g warfarin supplemented with 1.5 mg/g vitamin K1) to induce vascular calcification Concurrently, mice in the control group were fed a normal diet (Becht et al., 2016). Both groups of mice were maintained under standard conditions with regular changes in diet and water for a duration of 8 weeks. All animal procedures were approved by the Dalian Medical University Animal Care and Ethics Committee and were performed in accordance with the guidelines for the Care and Use of Laboratory Animals.

At the conclusion of the 8-week experimental period, the mice in each group were euthanized via cervical dislocation. Peripheral blood was collected and coronary artery tissues were carefully dissected. The collected coronary artery tissues were fixed in paraformaldehyde and embedded in paraffin for histological analysis.

### 2.10 Total RNA extraction and qRT-PCR

Total RNA was isolated from peripheral blood using the RNAeasy Animal RNA Extraction Kit (R0024, Beyotime Biotechnology, Shanghai, China) in accordance with the manufacturer’s instructions. Subsequently, quantitative real-time PCR (qRT-PCR) was conducted utilizing the AceQ qPCR SYBR Green Master Mix (Q131-02, Vazyme, Nanjing, China). The relative expression levels of PXDN were determined and calculated using the 2^−ΔΔCT^ method, with GAPDH serving as the internal reference gene. The primers used for qRT-PCR were as follows: PXDN (forward, 5′-GTT​CAG​CAT​GGC​TTG​ATG​GTG​G-3′; reverse, 3′-AGC​CTG​ACA​GGT​TGG​CGA​TGA​G-5′); GAPDH (forward, 5′-CAT​CAC​TGC​CAC​CCA​GAA​GAC​TG-3′; reverse, 3′-ATG​CCA​GTG​AGC​TTC​CCG​TTC​AG-5′). Standardization of data was performed on GAPDH expression.

### 2.11 Hematoxylin and eosin staining

Coronary artery tissues were subjected to fixation by immersion in 4% paraformaldehyde for a period of 24 h at room temperature. Subsequently, the fixed tissues were subjected to routine processing and subsequently embedded in paraffin blocks. Sections were then prepared using a microtome to achieve a thickness of 4 μm. These sections were subsequently subjected to staining with Hematoxylin and Eosin (H&E) in accordance with standard histological procedures, with a view to providing a comprehensive evaluation of the overall morphology of the coronary artery wall. The Olympus FSX100 microscope was utilized for microscopic examination and image acquisition of the stained sections.

### 2.12 Von kossa staining

Coronary artery tissues were fixed in 10% neutral buffered formalin for 6 h, routinely processed, paraffin-embedded, and sectioned at 4 μm. After deparaffinization and rehydration, sections were subjected to Von Kossa staining by incubation in 0.5%–2% silver nitrate under ultraviolet light exposure for 15–60 min, followed by treatment with 5% sodium thiosulfate to remove unreacted silver. Sections were subsequently counterstained with Hematoxylin and Eosin (H&E), dehydrated, cleared, and mounted. Calcified deposits appeared as black or brown-black areas under light microscopy and were imaged using an Olympus FSX100 microscope under standardized conditions.

### 2.13 Enzyme linked immunosorbent assay (ELISA)

Serum alkaline phosphatase (ALP) levels were quantified using an ELISA kit (P0321S, Beyotime, Shanghai, China). Plates were precoated with capture antibodies and incubated overnight at 4 °C. Following incubation, serum samples and standards were added to the wells and incubated at room temperature for 2 h. After washing the wells thoroughly, detection antibody was introduced and incubated for 2 h. Subsequently, HRP substrate was added and allowed to react for 30 min, followed by TMB substrate for 20 min in the dark. The reaction was terminated using stop solution, and absorbance was measured at 450 nm using a microplate reader. The data were analyzed to determine serum ALP concentrations based on the standard curve.

### 2.14 Calcium assay

The calcium content of coronary artery tissues was quantified using a commercial Ca^2+^ assay kit (TC1015, Leagene, China) according to the manufacturer’s instructions. Coronary artery tissues were homogenized to obtain protein lysates, which were centrifuged at 845 *g* for 15 min at 4 °C. The supernatants were collected and mixed with the working solution, followed by incubation at room temperature for 10 min. Absorbance was measured at 575 nm using a microplate reader, and relative calcium concentrations were calculated based on a standard curve.

### 2.15 Western blotting

Total protein was extracted from peripheral blood using a lysis buffer containing protease inhibitors. Protein concentration was determined using a BCA protein assay kit (P0010, Beyotime, Shanghai, China). Equal amounts of protein were separated by 10% SDS-PAGE (S8010, Solarbio, Beijing, China) and transferred to PVDF membranes (ISEQ00010, Millipore, USA). The membranes were blocked with 5% non-fat milk and then incubated overnight at 4 °C with primary antibodies against PXDN (1:1000, FNab10858, FineTest, Hubei, China) and GAPDH (1:500, ab8245, Abcam, Shanghai, China). Following the washing step, the membranes were then subjected to an incubation with HRP-conjugated secondary antibodies at room temperature for a period of 2 hours. Thereafter, the protein bands were subjected to visualization through the utilization of enhanced chemiluminescence (ECL) reagents (180-5001, Tanon, Shanghai, China). The quantification of band intensities was conducted utilizing ImageJ software, and the relative expression levels of target proteins were subjected to normalization to GAPDH.

### 2.16 Immunohistochemistry

The coronary artery tissue sections (4 μm) were deparaffinized in xylene and subsequently rehydrated through a graded series of ethanol solutions. Antigen retrieval was performed using a heat-induced epitope retrieval (HIER) method. In order to inhibit endogenous peroxidase activity, sections were exposed to 3% hydrogen peroxide for a period of 15 min at ambient temperature. Subsequently, sections were incubated overnight at 4 °C with a primary antibody against PXDN (1:100, FNab10858, FineTest, Hubei, China). Following three washes with Phosphate Buffered Saline (PBS), the sections were then subjected to an incubation with an HRP-conjugated secondary antibody (SP-9000, Zsbio, Beijing, China) for a period of 20 min at room temperature. Immunoreactivity was visualized using 3,3′-diaminobenzidine (DAB, AR1021, Boster, Wuhan, China) as the chromogen. Sections were then counterstained with hematoxylin, dehydrated, cleared, and mounted. The stained slides were then scanned using a whole-slide imaging system (SQS-40R, Shengqiang, Shenzhen, China). For the purpose of quantitative analysis, the histochemistry score (H-score) of PXDN-positive staining area was calculated using Aipathwell® software (Servicebio, Hubei, China).

### 2.17 Statistics and visualization

Statistical analyses for all graphs were conducted using the rstatix package (v0.7.2) and visualized with ggplot2 (v3.4.3), except where default statistical and visualization functions were employed. For comparing group differences, parametric tests such as Student’s t-test or Welch’s ANOVA were used when the data followed a normal distribution and met the assumptions of homogeneity. For non-normally distributed data, non-parametric tests such as the Wilcoxon rank-sum test or the Kruskal–Wallis test were performed, followed by Tukey’s post-hoc analysis for pairwise comparisons.

## 3 Results

### 3.1 Patients with coronary artery calcification exhibit significant metabolic reprogramming

**FIGURE 2:**
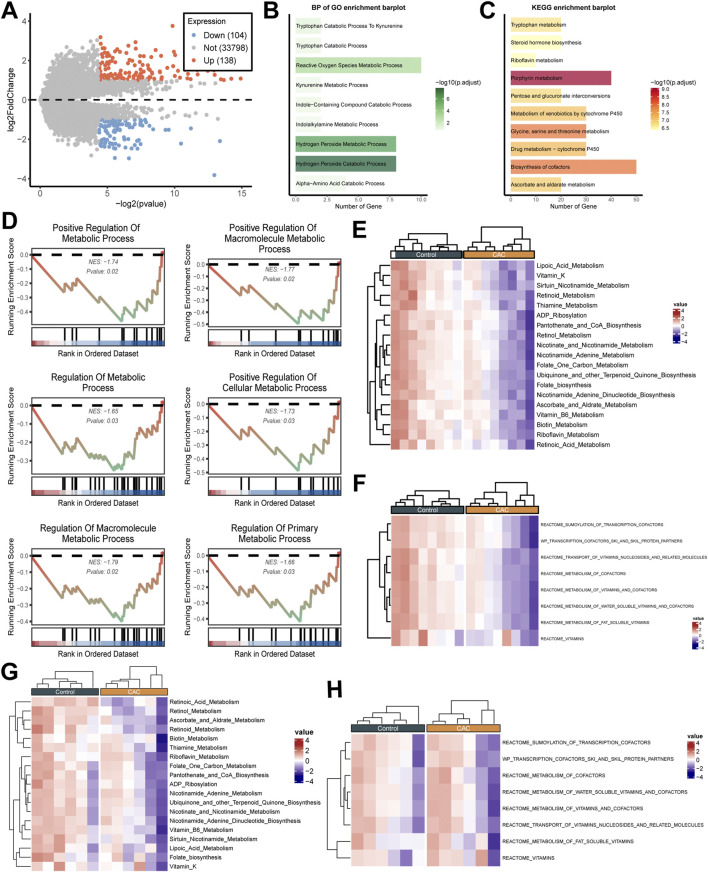
Functional enrichment analysis of differentially expressed genes and metabolic scoring of samples. **(A)** Volcano plot of differentially expressed genes (DEGs) showing upregulated genes (n = 138, red) and downregulated genes (n = 104, blue) relative to controls. **(B)** Gene ontology biological process (GO-BP) enrichment of DEGs. **(C)** Kyoto encyclopedia of genes and genomes (KEGG) pathway enrichment of DEGs. **(D)** Gene set enrichment analysis (GSEA) plots for metabolism pathways. **(E,F)** Heatmaps of metabolic scaled scores across samples in GSE58150 cohort. **(G,H)** Heatmaps of metabolic scaled scores across samples in GSE211752 cohort.

No samples met the exclusion criteria, and all were retained. In the GSE58150 dataset, 138 genes were found to be significantly upregulation in CAC, whilst 104 genes were found to be downregulation (*P < 0.05*) (Figure 2A; Supplementary Table S1). Subsequent ORA indicated 242 DEGs exhibited a strong correlation with metabolic processes, including “Reduced Oxygen Species Metabolic Process”, “Tryptophan Catabolic Process” and so on (*P < 0.05)* (Figure 2B). These genes were found to be significantly enriched in metabolic-related signaling pathways, such as “Retinol Metabolism”, “Arachidonic Acid Metabolism”, and “Biosynthesis of Secondary Metabolites” (*P < 0.05*) (Figure 2C). Concurrently, GSEA revealed that metabolic pathways, including “Positive Regulation of Metabolic Process”, “Positive Regulation of Cellular Metabolic Process”, and “Regulation of Metabolic Process”, exhibited significant inhibition in CAC (*P < 0.05*) (Figure 2D). Furthermore, in two independent datasets (GSE58150 and GSE211752), incorporating the metabolic feature gene set embedded in IOBR or metabolic-related genes from databases, consistently revealed that CAC exhibited lower metabolic scores than controls. This finding pertains to multiple distinct metabolic pathways, including “Vitamin A Metabolism”, “Lipid Acid Metabolism”, and “Cofactor Biosynthesis” (Figures 2E–H). In summary, metabolic-related signaling pathways are significantly suppressed in CAC, with markedly reduced metabolic scores, indicating significant metabolic reprogramming.

### 3.2 The central modules of the gene co-expression network are closely associated with metabolic reprogramming

**FIGURE 3:**
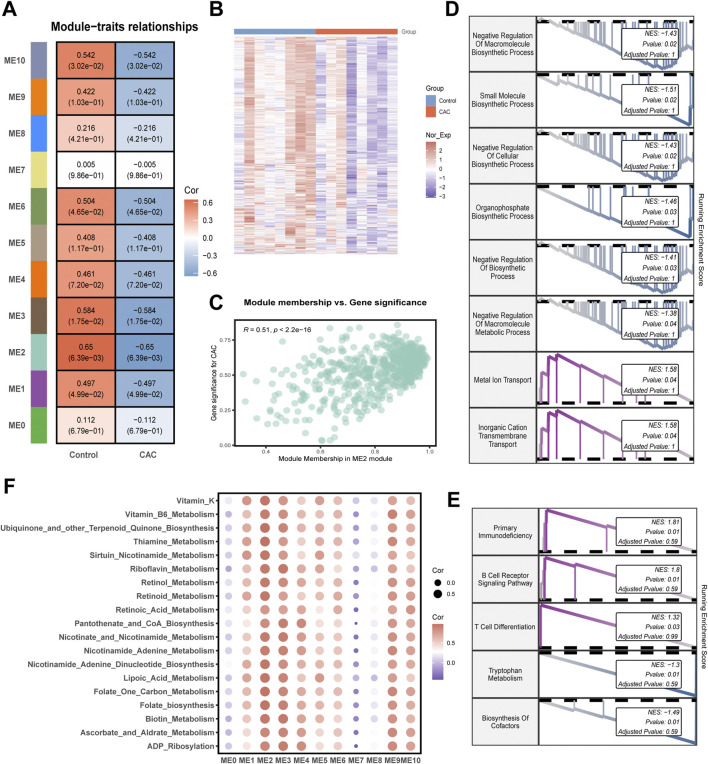
Co-expression modules, functional enrichment, and pathway associations in GSE58150 cohort. **(A)** Module–trait relationship heatmap showing correlations between weighted gene co-expression network modules and clinical groups. **(B)** Heatmap of ME2 module genes across control and CAC. **(C)** Scatter plot showing the relationship between module membership in ME2 and gene significance. **(D)** Gene set enrichment analysis (GSEA) plots for Gene ontology biological process (GO-BP). **(E)** GSEA plots for Kyoto encyclopedia of genes and genomes (KEGG) pathway enrichment. **(F)** Heatmap of correlations between module eigengenes (ME0–ME10) and metabolism pathways.

As the power value increases, the scaling index (R^2^) of the gene co-expression network significantly rises, reaching the threshold of 0.85 when the power value is 11. At this point, the average connectivity of the network also decreases to a stable range, enabling the network to be further partitioned into 11 gene modules (Figure 3A; Supplementary Figures S1A, B). Among these, the ME2 module exhibited the highest absolute correlation coefficient (0.65) with patient classification, indicating its close association with the occurrence and development of CAC. Subsequent stability analysis of the modules revealed that the Zsummary corresponding to the ME2 module was significantly greater than 10, and its median rank for module conservation was also below the threshold (Supplementary Figures S1C, D). This demonstrates that the current gene module is highly conserved and possesses the strongest biological stability. Additionally, results indicate that the module membership in ME2 module shows a significant positive correlation with gene significance for CAC (R = 0.51, *P < 0.001*) (Figure 3C). Concurrently, ME2 module genes exhibit significantly downregulated expression in CAC patients (Figure 3B), suggesting that downregulation of ME2 module genes promotes the onset and progression of CAC. In subsequent GSEA analysis of genes in the ME2 module, it was found that they were closely associated with metabolic processes such as “Negative Regulation of Macromolecule Biosynthetic Process”, “Small Molecule Biosynthetic Process”, and “Organophosphate Biosynthetic Process” (Figure 3D), and were significantly enriched in metabolic-related pathways such as “Nitrogen Metabolism” and “Tryptophan Metabolism” (Figure 3E). Additionally, the ME2 module exhibited the strongest positive correlation with metabolic scores (Figure 3F), indicating that high expression of ME2 module genes correlates with enhanced metabolic activity, while low expression is associated with reduced pathway activity. In short, the overall downregulated expression pattern of ME2 module genes in CAC patients is consistent with the reduced metabolic scores observed in the same cohort, as well as the significant suppression of metabolism-related pathways identified by GSEA. These findings collectively support the presence of metabolic reprogramming in CAC and further highlight the biological relevance of the ME2 module in disease progression.

### 3.3 Integrating protein-protein interaction networks and machine learning to identify key genes

**FIGURE 4:**
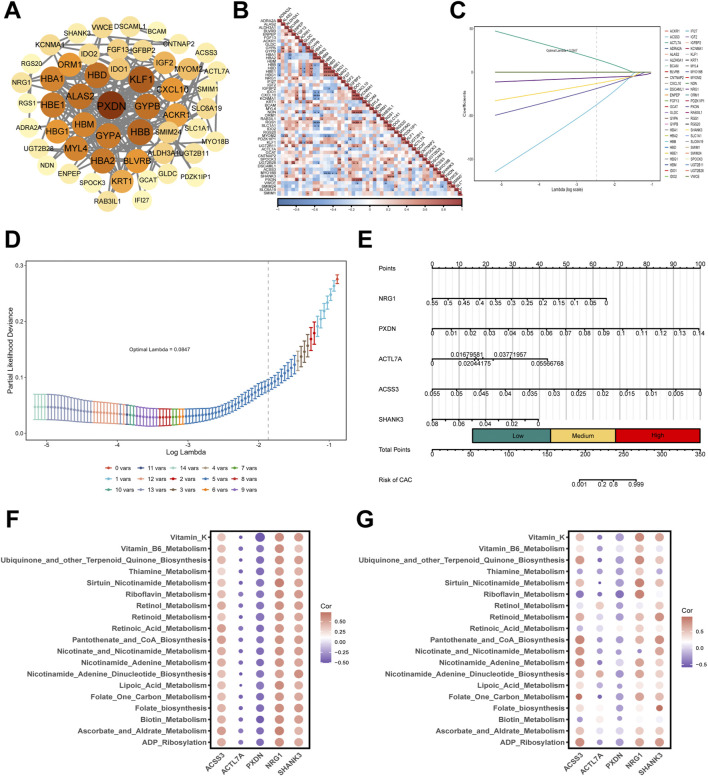
Identification of hub genes, construction of a risk model, and metabolic pathway correlations in CAC. **(A)** Protein–protein interaction (PPI) network highlighting hub genes within the significant module. **(B)** Correlation heatmap showing expression associations among candidate genes. **(C)** LASSO coefficient profiles of candidate genes across different values of the regularization parameter λ. **(D)** Cross-validation plot for tuning parameter selection in the LASSO regression. **(E)** Nomogram integrating five genes (NRG1, PXDN, ACTL7A, ACS53, and SHANK3) to predict individual risk of CAC. **(F)** Bubble plots showing correlations between CAC-associated genes and metabolism pathways in GSE58150 cohort. Positive associations (red) and negative associations (blue), with dot size reflecting correlation strength. **(G)** Bubble plots showing correlations between CAC-associated genes and metabolism pathways in GSE211752 cohort.

In the interaction analysis of ME2 module genes, a gene-based screening process was undertaken, resulting in the construction of a protein interaction network comprising 52 genes (Figure 4A). The analysis identified PXDN, KLF1, and HBB as being central to the network, exhibiting pronounced associations with other genes (Figure 4A). Concurrently, the expression correlation analysis revealed a substantial positive correlation between PXDN and KLF1 with the majority of genes, thereby substantiating their pivotal functions (Figure 4B). In the ensuing Lasso regression, it was ascertained that a model formulated using the five genes NRG1, PXDN, ACTL7A, ACSS3, and SHANK3 exhibited the capacity to efficaciously predict the likelihood of individuals developing CAC (Figures 4C,D).

**FIGURE 5:**
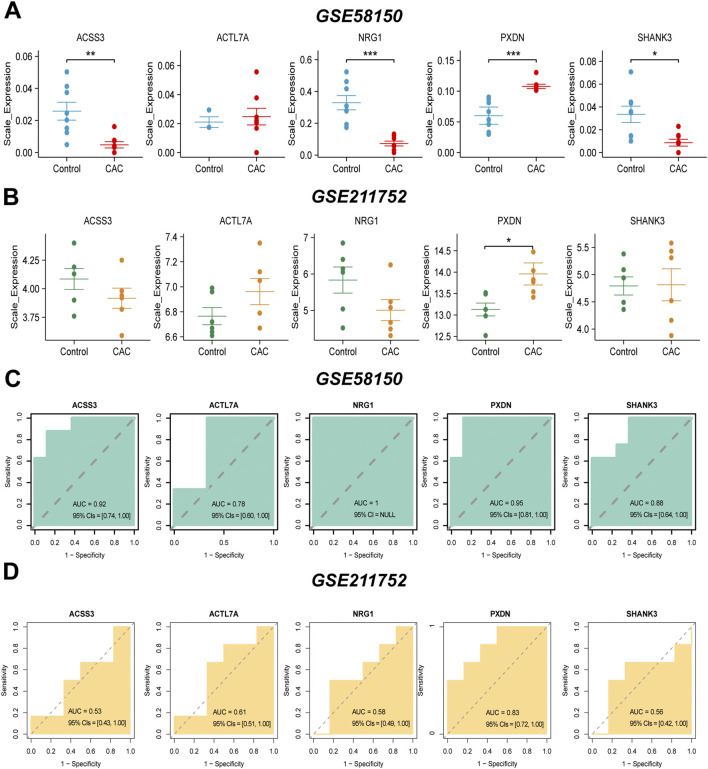
Validation of CAC-associated genes and their diagnostic performance in independent cohorts. **(A)** Expression levels of ACSS3, ACTL7A, NRG1, PXDN, and SHANK3 in the GSE58150 dataset. **(B)** Expression levels of the same five genes in the GSE211752 dataset. **(C)** Receiver operating characteristic (ROC) curves for the five genes in GSE58150. **(D)** ROC curves for the five genes in GSE211752. Wilcoxon test was used in **(A,B)**. *P < 0.05, **P ≤ 0.01, ***P ≤ 0.001.

To rigorously evaluate the discriminatory performance of candidate genes, ROC analyses were conducted in both the discovery cohort (GSE58150) and the independent validation cohort (GSE211752). In the discovery cohort, several genes demonstrated strong discriminative capacity, with PXDN achieving an AUC of 0.95 (95% CIs: 0.81–1.00), ACSS3 0.92, SHANK3 0.88, ACTL7A 0.78, and NRG1 1.00 (Figures 5A–D). However, given the limited sample size, bootstrap resampling (R = 1000) was performed to assess the stability of these estimates. Bootstrap analysis showed minimal bias and relatively small standard errors for PXDN in both cohorts (bias = −0.00056, SE = 0.03175 in GSE58150; bias = 0.00238, SE = 0.01664 in GSE211752), indicating stable performance. In contrast, other genes exhibited larger standard errors and greater variability in the validation cohort. Notably, in GSE211752, ACSS3 (AUC = 0.53), ACTL7A (AUC = 0.61), NRG1 (AUC = 0.58), and SHANK3 (AUC = 0.56) showed limited discriminative ability and unstable bootstrap estimates (Supplementary Table S2). Permutation testing further confirmed that only PXDN retained statistical significance in both datasets (*P < 0.01*), whereas the remaining genes did not demonstrate performance significantly better than random classification in the validation cohort (Supplementary Table S3). Collectively, these results indicate that although multiple genes exhibited high AUC values in the discovery cohort, PXDN was the only candidate demonstrating reproducible and statistically robust diagnostic performance across independent datasets. These findings support PXDN as the most stable and reliable circulating biomarker candidate for CAC.

### 3.4 Patients with coronary artery calcification exhibit abundant immune infiltration

**FIGURE 6:**
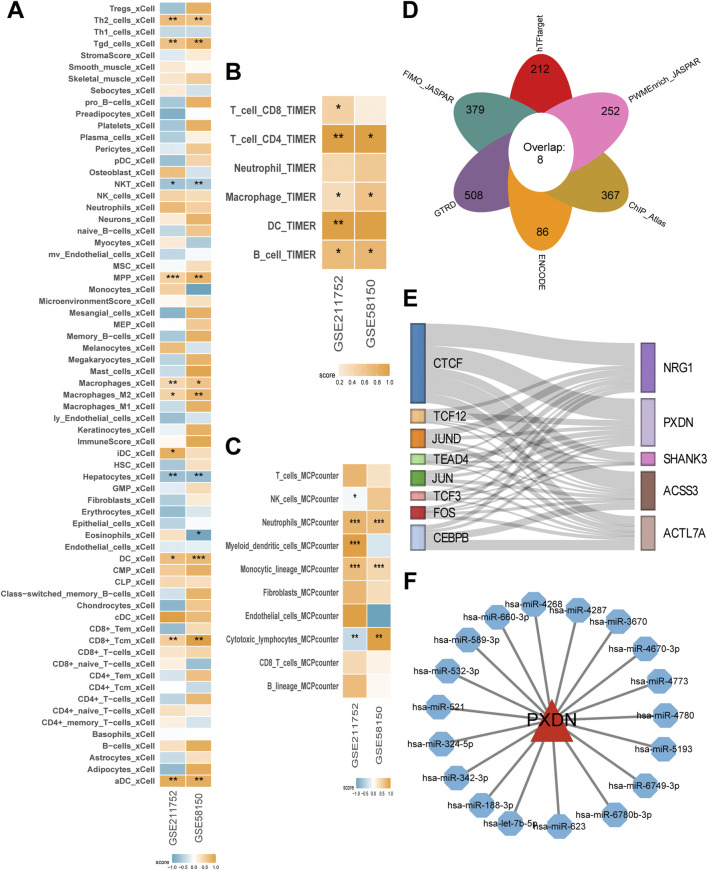
Immune infiltration patterns, regulatory networks, and PXDN-associated interactions in CAC. **(A-C)** Heatmap showing the enrichment scores of various immune and stromal cell types in control and CAC across GSE58150 and GSE211752 cohorts based on xCell, TIMER and MCP-counter. The color intensity represents the degree of enrichment. **(D)** Venn diagram of transcription factors predicted by multiple databases (JASPAR, ChIP Atlas, ENCODE, TRRUST, and others), identifying eight overlapping candidates. **(E)** Regulatory network connecting key transcription factors with five CAC-associated genes. **(F)** Predicted microRNA–gene interaction network for PXDN, showing multiple miRNAs potentially regulating PXDN expression. *P < 0.05, **P ≤ 0.01, ***P ≤ 0.001.

In order to ascertain whether there are discrepancies in the IME between CAC and the control, three distinct algorithms were utilized to quantify the cell types present in the microenvironment. The findings demonstrated that, in both the GSE58150 and GSE211752 datasets, compared with the control, the infiltration abundance of macrophages, monocytes, dendritic cells, and T cells in the microenvironments of CAC was significantly elevated, indicating high immune activity (Figures 6A–C). In contrast, the control exhibited abundant infiltration of non-immune cells, including endothelial cells, mesangial cells, and fibroblasts, with reduced immune activity (Figures 6A–C).

Subsequently, integration of the content of six independent databases led to the extraction of eight highly reliable transcription factors for NRG1, PXDN, ACTL7A, ACSS3, and SHANK3 (Figure 6D). Among them, PXDN was associated with all eight transcription factors, indicating that they may all participate in the expression regulation of PXDN (Figure 6E). Additionally, in the network of microRNA-mRNA, PXDN was closely associated with 18 microRNAs, including hsa-miR-4268 and hsa-miR-4287, suggesting that its expression may also be influenced by these microRNAs (Figure 6F).

### 3.5 PXDN, a circulating biomarker for coronary artery calcification

**FIGURE 7:**
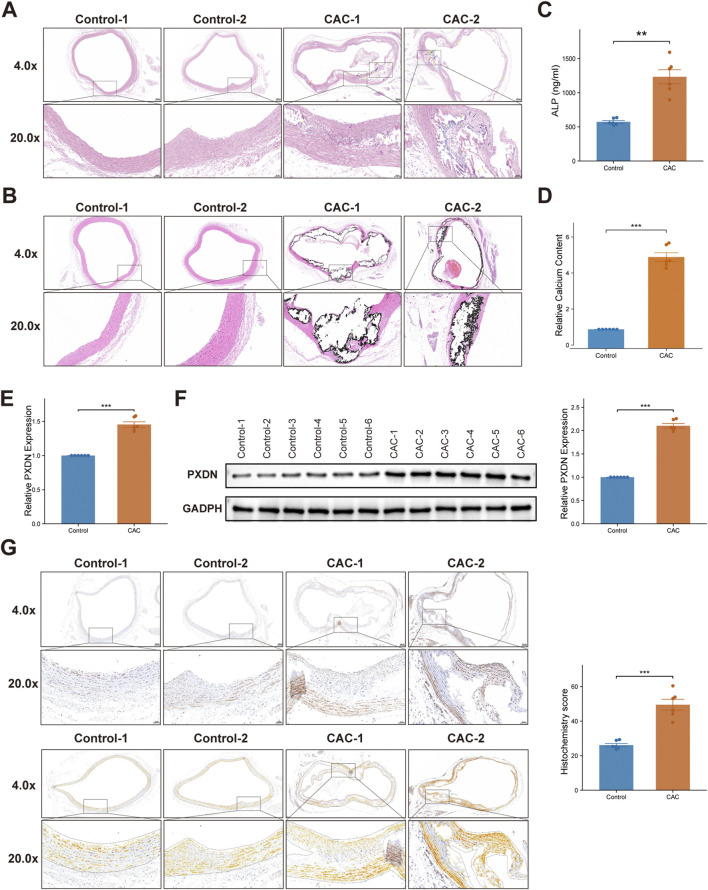
Histological and molecular validation of PXDN upregulation in CAC. **(A)** Representative hematoxylin and eosin (HE) staining images of coronary artery tissue cross-sections from control and CAC groups, shown at ×4 and ×20 magnification. **(B)** Representative von kossa (VK) staining images of coronary artery tissue cross-sections from control and CAC groups, shown at ×4 and ×20 magnification. **(C)** Serum ALP levels were significantly elevated in CAC compared with controls. **(D)** Quantitative calcium content was significantly elevated in CAC compared with controls. **(E)** qRT-PCR analysis showing higher PXDN mRNA expression in CAC tissues compared with controls. **(F)** Western blot validation of PXDN expression with densitometric quantification normalized to GAPDH. **(G)** Representative immunohistochemical (IHC) staining (upper panels) and scanning staining (lower panels) of PXDN in coronary artery tissue, and semiquantitative H-score analysis (right panel). Wilcoxon test was used in **(C–F)**. *P < 0.05, **P ≤ 0.01, ***P ≤ 0.001.

Histological staining of the coronary arteries revealed that the vascular tissue structure in control mice was unremarkable, with an intact endothelial layer in the intima and no obvious hyperplasia. The vascular wall layers were clearly organized, with elastic laminae and smooth muscle cells arranged in a compact and regular pattern within the media (Figure 7A). In contrast, coronary arteries from CAC mice exhibited marked structural disruption. Extensive necrotic areas were observed within the vascular wall, particularly involving the medial layer, where the structures of elastic fibers and smooth muscle cells appeared blurred and disorganized. Numerous necrotic cellular debris and amorphous eosinophilic material (yellow arrows) were present, accompanied by focal calcium salt deposition (orange arrows) and multiple ruptures of elastic laminae (cyan arrows). The intima occasionally showed mild connective tissue proliferation (green arrows) and sporadic brownish-yellow pigment deposition (brown arrows). Minimal inflammatory cell infiltration, predominantly lymphocytes (blue arrows), was observed in the intima and adventitia, and occasional hemorrhagic changes were noted in the adventitia (pink arrows) (Figure 7A).

To further verify vascular calcification, von kossa (VK) staining was performed. In CAC mice, prominent black deposits indicating calcium phosphate accumulation were detected within the medial layer of the coronary arterial wall, whereas little to no staining was observed in control mice (Figure 7B). The calcified regions largely overlapped with the areas of medial structural disruption observed in H&E staining, suggesting that the calcification in this model primarily occurred in the vascular media rather than the intima. Moreover, compared with controls, serum alkaline phosphatase (ALP) levels in CAC mice were significantly elevated (*P < 0.01*) (Figure 7C). Consistently, quantitative calcium content analysis demonstrated that the calcium levels in coronary arteries of CAC mice were significantly higher than those of control mice (*P < 0.01*) (Figure 7D), further confirming the presence of coronary artery calcification. The qRT-PCR and Western blotting results demonstrated that the expression of PXDN in the peripheral blood of CAC mice was significantly increased at both the transcriptional and protein levels (*P < 0.001*), which was consistent with the results obtained from publicly available datasets (Figures 7E,F). Furthermore, immunohistochemical analysis revealed that the histochemistry score for PXDN in the coronary arteries of CAC mice was significantly higher compared with the control (*P < 0.001*), indicating induced PXDN expression (Figure 7G).

## 4 Discussion

Coronary artery calcification (CAC) is widely recognized as an important pathological feature of coronary artery disease and a strong predictor of future cardiovascular events (Martin et al., 2025; Greenland et al., 2010; Detrano et al., 2008; Polonsky et al., 2010; Leong et al., 2017). Although previous studies have identified several biological processes involved in CAC, including phosphate metabolism, vascular smooth muscle cell osteogenic differentiation, and macrophage polarization (Jono et al., 2000; Jaume et al., 2010; Tsuruta et al., 2008; Floege et al., 2010; Wu et al., 2014; Makows et al., 2020; Gordon and Plüddemann, 2017; Chen et al., 2019; Chen et al., 2024), the broader metabolic landscape underlying CAC remains incompletely understood. In particular, whether CAC is associated with coordinated alterations across multiple metabolic pathways has not been systematically investigated.

In the present study, we performed a transcriptomic analysis to explore metabolic alterations associated with CAC. Differential expression analysis identified 242 genes that were significantly dysregulated in CAC samples. Functional enrichment analyses suggested that these genes were closely associated with several metabolism-related pathways, including oxygen free radical metabolism, tryptophan metabolism, retinol metabolism, and arachidonic acid metabolism. Consistently, metabolic scoring analysis revealed that several metabolism-related signatures were significantly reduced in CAC individuals compared with controls. Furthermore, weighted gene co-expression network analysis identified the ME2 module as the gene cluster most strongly associated with CAC status. Notably, genes within this module were significantly downregulated in CAC and were enriched in metabolic signaling pathways. Together, these results suggest that CAC is accompanied by widespread transcriptional changes involving multiple metabolic processes. Circulating biomarkers represent an attractive strategy for improving early detection and risk stratification of cardiovascular diseases. Compared with invasive procedures such as coronary angiography or imaging modalities like computed tomography, blood-based biomarkers offer advantages in accessibility and feasibility for longitudinal monitoring. However, currently available biomarkers for coronary artery disease often lack sufficient sensitivity or specificity, particularly during the early stages of disease (Wong and Tse, 2021; Mansour et al., 2025; Nazir et al., 2025). Increasing evidence from other diseases indicates that metabolic alterations can serve as a valuable source for biomarker discovery (Xu et al., 2025; Li B. et al., 2025). For example, alterations in tryptophan–kynurenine metabolites have been shown to correlate with prognosis in colorectal cancer patients (Xu et al., 2025), while metabolic signatures have also been used to construct diagnostic models for brainstem glioma (Hoffmann et al., 2024). Moreover, genes regulating metabolic pathways frequently reflect underlying metabolic states and have been widely used as biomarkers for disease diagnosis and prognosis (Zou et al., 2024; Damerell et al., 2025; Gu et al., 2024).

Based on these considerations, we sought to identify metabolism-related genes with potential diagnostic value in CAC. By integrating protein–protein interaction network analysis with Lasso regression, we identified five candidate genes (NRG1, PXDN, ACTL7A, ACSS3, and SHANK3) associated with CAC diagnosis. Among these candidates, PXDN was the only gene that showed consistent upregulation across independent datasets and demonstrated strong diagnostic performance, with AUC values of 0.95 and 0.83. In addition, PXDN expression showed a significant negative correlation with metabolic scores. Importantly, experimental validation further demonstrated that PXDN expression was markedly elevated in the peripheral blood of CAC mice at both the transcriptional and protein levels, and PXDN immunostaining was significantly increased in coronary artery tissues. Collectively, these findings suggest that PXDN may represent a promising circulating biomarker associated with CAC. PXDN encodes peroxidasin, a member of the peroxidase–epoxygenase family that catalyzes oxidative reactions using hydrogen peroxide as a substrate (Li K. et al., 2025; Puurand et al., 2024; Dakal et al., 2024). Through this process, PXDN can generate several reactive oxidizing species, including hypochlorous acid, hypobromous acid, and hypothiocyanous acid, which have been implicated in oxidative stress–related tissue injury. Previous studies have reported that PXDN-mediated oxidation of ApoE can impair lipid efflux and promote the development of atherosclerosis (Liu et al., 2025). In addition, PXDN has been linked to oxidative stress responses in several cardiovascular contexts, including myocardial ischemia–reperfusion injury and endothelial cell apoptosis (Dakal et al., 2024; Cheng and Shi, 2022; Cheng et al., 2008).

In the present study, increased PXDN expression was observed in both peripheral blood and coronary artery tissues of CAC mice. Combined with the known biochemical function of PXDN, these findings raise the possibility that PXDN-related oxidative processes may be involved in vascular injury and metabolic alterations associated with CAC. Oxidative stress is known to influence cellular metabolism through multiple mechanisms. For example, sustained oxidative stress can disrupt mitochondrial dynamics, impair oxidative phosphorylation, and alter cellular metabolic flux (Ma et al., 2013; Yang et al., 2013; Nabeebaccus et al., 2011). Furthermore, vascular injury accompanied by oxidative stress may promote inflammatory responses and immune cell infiltration, which can further influence metabolic pathways within vascular tissues (Bedard and Krause, 2007; Yan et al., 2023; Steven et al., 2019). Therefore, it is plausible that PXDN-associated oxidative activity may contribute to the metabolic changes observed in CAC.

Several limitations of the present study should be acknowledged. First, the identification of metabolic alterations in CAC was primarily based on transcriptomic analyses, and direct metabolomic measurements were not performed. Integrating metabolomic profiling with transcriptomic data in future studies would provide more direct evidence of metabolic reprogramming. Second, although PXDN expression was experimentally validated in both public datasets and animal models, functional experiments were not conducted to determine whether PXDN directly contributes to vascular calcification, oxidative stress, or metabolic remodeling. Therefore, the proposed relationship between PXDN, oxidative stress, and metabolic alterations should be interpreted as a potential mechanistic hypothesis rather than a demonstrated causal pathway. Future studies incorporating PXDN perturbation in vascular or immune cells, as well as measurements of oxidative stress markers, mitochondrial function, and metabolic flux, will be required to clarify its biological role in CAC. Third, the bioinformatic analyses relied on publicly available datasets, which may introduce potential biases related to sample heterogeneity or data processing. In addition, the sample size included in the present study was relatively limited, and larger prospective cohorts will be necessary to validate the robustness and clinical applicability of our findings. Despite these limitations, our results consistently demonstrate that PXDN is significantly upregulated in CAC and may serve as a promising circulating biomarker reflecting disease-associated metabolic alterations. These findings provide a valuable foundation for future mechanistic investigations and for the development of non-invasive biomarkers for early detection and risk stratification of coronary artery calcification.

## 5 Conclusion

This study revealed that individuals with coronary artery calcification exhibit widespread transcriptional alterations in metabolism-related pathways, indicating the presence of metabolic reprogramming signatures. Through integrative bioinformatics analyses and experimental validation, PXDN was identified as a circulating biomarker candidate associated with CAC. Although the mechanistic link between PXDN, oxidative stress, and metabolic alterations requires further investigation, our findings provide a foundation for future studies on metabolism-related biomarkers and contribute to improved risk stratification and non-invasive assessment of coronary artery calcification.

## Data Availability

The original contributions presented in the study are included in the article/Supplementary Material, further inquiries can be directed to the corresponding authors.

## Glossary

ACTL7A: Actin Alpha Cardiac Muscle 7
ACSS3: Acyl-CoA Synthetase Short-Chain Family Member 3
AUC: Area Under the Curve
BCA: Bicinchoninic Acid
BNP: B-type Natriuretic Peptide
CAC: Coronary Artery Calcification
CAD: Coronary Artery Disease
CIMLR: Cancer Integration via Multikernel Learning
CIs: Confidence Intervals
CT: Computed Tomography
DAB: 3,3′-Diaminobenzidine
DEGs: Differentially Expressed genes
ELISA: Enzyme-Linked Immunosorbent Assay
ECL: Enhanced Chemiluminescence
FDR: False Discovery Rate
GEO: Gene Expression Omnibus
GSEA: Gene Set Enrichment Analysis
GO: Gene Ontology
H2O2: Hydrogen Peroxide
H&E: Hematoxylin and Eosin
HOCl: Hypochlorous Acid
HOBr: Hypobromous Acid
HOSCN: Hypothiocyanous Acid
HRP: Horseradish Peroxidase
H-score: Histochemistry Score
IOBR: Immuno-Oncology Biological Research
KEGG: Kyoto Encyclopedia of Genes and Genomes
LASSO: Least Absolute Shrinkage and Selection Operator
MAD: Median Absolute Deviation
MCPcounter: Microenvironment Cell Populations-counter
miRNA: MicroRNA
NRG1: Neuregulin 1
ORA: Over-Representation Analysis
PBS: Phosphate Buffered Saline
PCA: Principal Component Analysis
PPI: Protein-Protein Interaction
PXDN: Peroxidasin
PTH: Parathyroid Hormone
qRT-PCR: Quantitative Real-Time Polymerase Chain Reaction
RMA: Robust Multi-array Average
RNA-seq: RNA Sequencing
ROC: Receiver Operating Characteristic
SHANK3: SH3 and Ankyrin Domain Containing 3
SDS-PAGE: Sodium Dodecyl Sulfate Polyacrylamide Gel Electrophoresis
SPF: Specific Pathogen-Free
ssGSEA: Single-Sample Gene Set Enrichment Analysis
TF: Transcription Factor
TMB: 3,3′,5,5′-Tetramethylbenzidine
TIMER: Tumor Immune Estimation Resource
TOM: Topological Overlap Matrix
TPM: Transcripts Per Million
WGCNA: Weighted Gene Co-expression Network Analysis
XCell: Cell Type Enrichment Analysis
