# Supplementary material for: Decoding coronary artery calcification: metabolic reprogramming features and a promising circulating biomarker PXDN
Source: Front Cell Dev Biol. 2026 Apr 10;14:1714146. doi: 10.3389/fcell.2026.1714146 (PMC13106572; doi:10.3389/fcell.2026.1714146)
Supplement: Supplementary file 2 [file DataSheet1.docx]

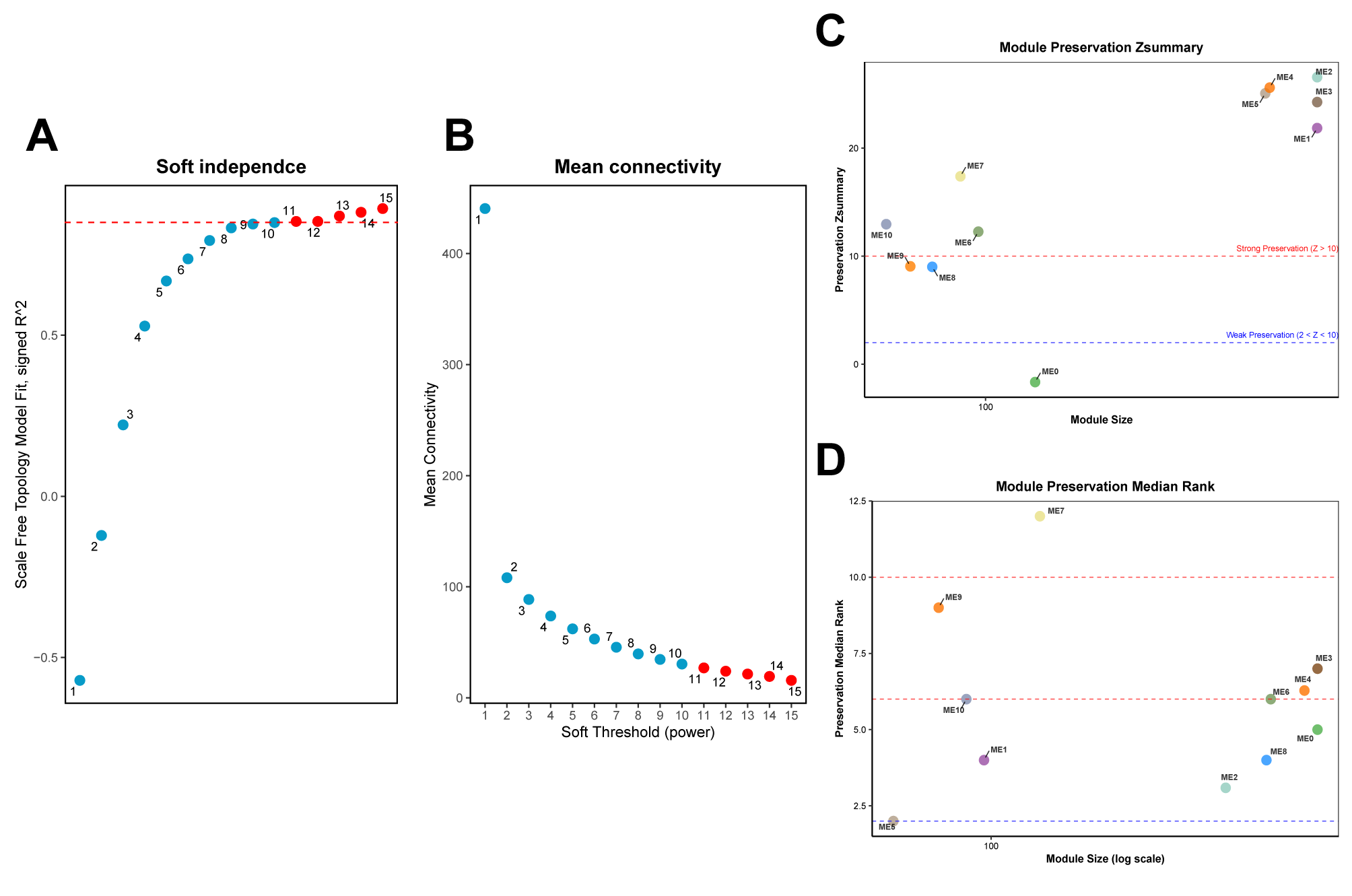


**Supplementary Figure 1.** Selection of soft-thresholding power and module preservation analysis in weighted gene co-expression network analysis (WGCNA). (A) Analysis of the scale-free topology fit index (R²) for different soft-thresholding powers. The red dashed line indicates the threshold of R² = 0.9, used to determine the optimal soft-thresholding power for network construction. (B) Mean connectivity of the network under different soft-thresholding powers, showing the decrease in connectivity as the power increases. (C) Module preservation summary plot displaying the relationship between module size and Zsummary statistics. Modules with Zsummary > 10 indicate strong preservation, while those with 2 < Zsummary ≤ 10 indicate weak to moderate preservation. (D) Module preservation median rank analysis.
